# Deep Patient: An Unsupervised Representation to Predict the Future of Patients from the Electronic Health Records

**DOI:** 10.1038/srep26094

**Published:** 2016-05-17

**Authors:** Riccardo Miotto, Li Li, Brian A. Kidd, Joel T. Dudley

**Affiliations:** 1 Department of Genetics and Genomic Sciences, Icahn School of Medicine at Mount Sinai, New York, NY, USA; 2 Harris Center for Precision Wellness, Icahn School of Medicine at Mount Sinai, New York, NY, USA; 3 Icahn Institute for Genomics and Multiscale Biology, Icahn School of Medicine at Mount Sinai, New York, NY, USA

## Abstract

Secondary use of electronic health records (EHRs) promises to advance clinical research and better inform clinical decision making. Challenges in summarizing and representing patient data prevent widespread practice of predictive modeling using EHRs. Here we present a novel unsupervised deep feature learning method to derive a general-purpose patient representation from EHR data that facilitates clinical predictive modeling. In particular, a three-layer stack of denoising autoencoders was used to capture hierarchical regularities and dependencies in the aggregated EHRs of about 700,000 patients from the Mount Sinai data warehouse. The result is a representation we name “deep patient”. We evaluated this representation as broadly predictive of health states by assessing the probability of patients to develop various diseases. We performed evaluation using 76,214 test patients comprising 78 diseases from diverse clinical domains and temporal windows. Our results significantly outperformed those achieved using representations based on raw EHR data and alternative feature learning strategies. Prediction performance for severe diabetes, schizophrenia, and various cancers were among the top performing. These findings indicate that deep learning applied to EHRs can derive patient representations that offer improved clinical predictions, and could provide a machine learning framework for augmenting clinical decision systems.

A primary goal of precision medicine is to develop quantitative models for patients that can be used to predict health status, as well as to help prevent disease or disability. In this context, electronic health records (EHRs) offer great promise for accelerating clinical research and predictive analysis^1^. Recent studies have shown that secondary use of EHRs has enabled data-driven prediction of drug effects and interactions^2^, identification of type 2 diabetes subgroups^3^, discovery of comorbidity clusters in autism spectrum disorders^4^, and improvements in recruiting patients for clinical trials^5^. However, predictive models and tools based on modern machine learning techniques have not been widely and reliably used in clinical decision support systems or workflows^6^,^7^,^8^,^9^.

EHR data is challenging to represent and model due to its high dimensionality, noise, heterogeneity, sparseness, incompleteness, random errors, and systematic biases^7^,^10^,^11^. Moreover, the same clinical phenotype can be expressed using different codes and terminologies. For example, a patient diagnosed with “type 2 diabetes mellitus” can be identified by laboratory values of hemoglobin A1C greater than 7.0, presence of 250.00 ICD-9 code, “type 2 diabetes mellitus” mentioned in the free-text clinical notes, and so on. These challenges have made it difficult for machine learning methods to identify patterns that produce predictive clinical models for real-world applications^12^.

The success of predictive algorithms largely depends on feature selection and data representation^12^,^13^. A common approach with EHRs is to have a domain expert designate the patterns to look for (i.e., the learning task and the targets) and to specify clinical variables in an ad-hoc manner^7^. Although appropriate in some situations, supervised definition of the feature space scales poorly, does not generalize well, and misses opportunities to discover novel patterns and features. To address these shortcomings, data-driven approaches for feature selection in EHRs have been proposed^14^,^15^,^16^. A limitation of these methods is that patients are often represented as a 2-dimensional vector composed by all the data descriptors available in the clinical data warehouse. This representation is sparse, noisy, and repetitive, which makes it not suitable for modeling the hierarchical information embedded or latent in EHRs.

Unsupervised feature learning attempts to overcome limitations of supervised feature space definition by automatically identifying patterns and dependencies in the data to learn a compact and general representation that make it easier to automatically extract useful information when building classifiers or other predictors. Despite the success of feature learning with text, multimedia, and marketing^12^, as well as the rising popularity of deep learning^17^ (i.e., learning based on hierarchies of neural networks), these techniques have not been used broadly with EHR data. Here we show that unsupervised deep feature learning applied to pre-process patient-level aggregated EHR data results in representations that are better understood by the machine and significantly improve predictive clinical models for a diverse array of clinical conditions.

This paper presents a novel framework we call “deep patient” to represent patients by a set of general features, which are inferred automatically from a large-scale EHR database through a deep learning approach. Specifically, a deep neural network composed of a stack of denoising autoencoders was used to process EHRs in an unsupervised manner that captured stable structures and regular patterns in the data, which, grouped together, compose the deep patient representation. Deep patient is domain free (i.e., not related to any specific task since learned over a large multi-domain dataset), does not require any additional human effort, and can be easily applied to different predictive applications, both supervised and unsupervised. To prove the effectiveness of the proposed representation we apply deep patient to predict patient future diseases and show that the deep patient consistently outperforms original EHR representations as well as common (shallow) feature learning models in a large-scale real world data experiment.

## Material and Methods

This section presents the deep patient method and describes the pipeline implemented to evaluate the benefits of this representation in the task of predicting future diseases.

### Deep Patient Representation

**Figure 1:**
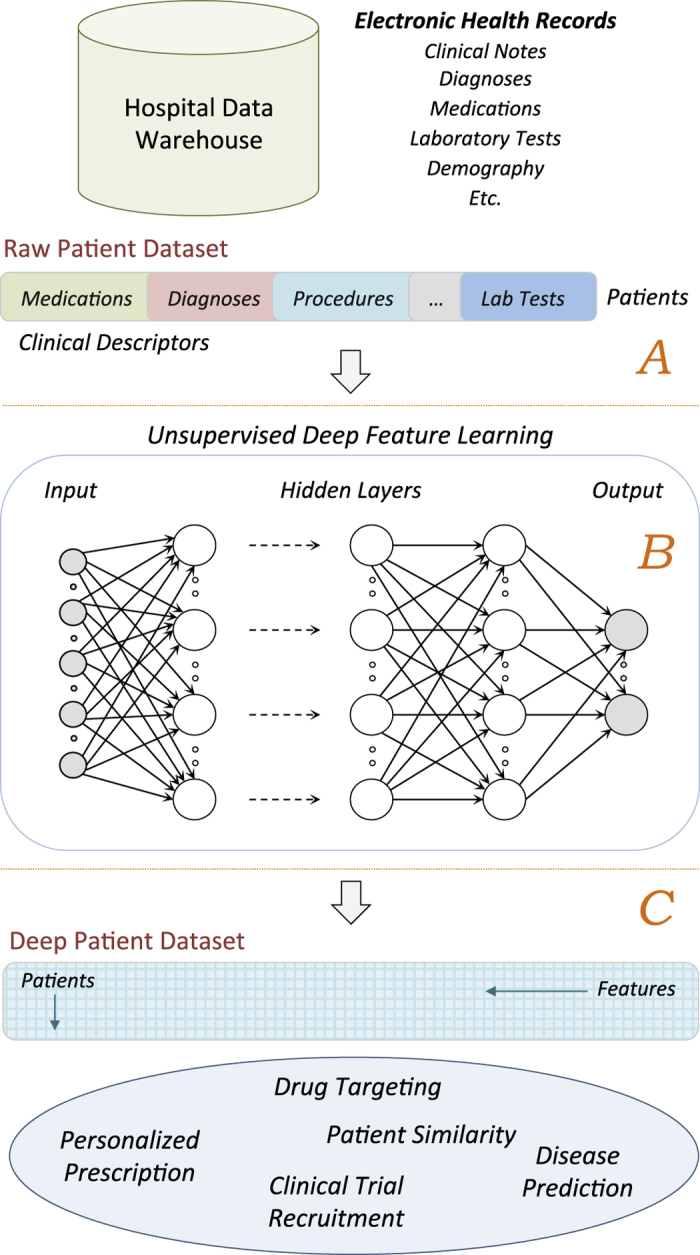
Conceptual framework used to derive the deep patient representation through unsupervised deep learning of a large EHR data warehouse. (**A**) Pre-processing stage to obtain raw patient representations from the EHRs. (**B**) The raw representations are modeled by the unsupervised deep architecture leading to a set of general and robust features. (**C**) The deep features are applied to the entire hospital database to derive patient representations that can be applied to a number of clinical tasks.

Figure 1 shows the high-level conceptual framework to derive the deep patient representation. EHRs are first extracted from the clinical data warehouse, pre-processed to identify and normalize clinically relevant phenotypes, and grouped in patient vectors (i.e., raw representation, Fig. 1A). Each patient can be described by just a single vector or by a sequence of vectors computed in, e.g., predefined temporal windows. The collection of vectors obtained from all the patients is used as input of the feature learning algorithm to discover a set of high level general descriptors (Fig. 1B). Every patient in the data warehouse is then represented using these features and such deep representation can be applied to different clinical tasks (Fig. 1C).

**Figure 2:**
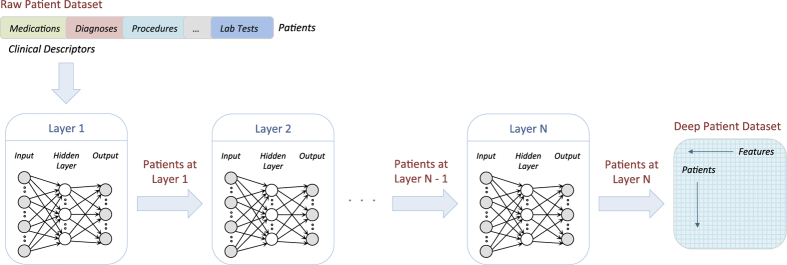
Diagram of the unsupervised deep feature learning pipeline to transform a raw dataset into the deep patient representation through multiple layers of neural networks. Each layer of the neural network is trained to produce a higher-level representation from the result of the previous layer.

We derived the patient representation using a multi-layer neural network in a deep learning architecture (i.e., deep patient). Each layer of the network is trained to produce a higher-level representation of the observed patterns, based on the data it receives as input from the layer below, by optimizing a local unsupervised criterion (Fig. 2). Every level produces a representation of the input pattern that is more abstract than the previous level because it is obtained by composing more non-linear operations. This process is loosely analogous to neuroscience models of cognition that hierarchically combine lower-level features to a unified and compact representation. The last network of the chain outputs the final patient representation.

### Denoising Autoencoders

We implemented our framework using a stack of denoising autoencoders (SDA), which are independently trained layer by layer; all the autoencoders in the architecture share the same structure and functionalities^18^. Briefly, an autoencoder takes an input 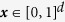 and first transforms it (with an *encoder*) to a hidden representation 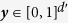
through a deterministic mapping:


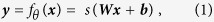


parameterized by 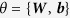, where 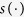 is a non-linear transformation (e.g., sigmoid, tangent) named “activation function”, ***W*** is a weight coefficient matrix, and ***b*** is a bias vector. The latent representation ***y*** is then mapped back (with a *decoder*) to a reconstructed vector 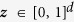, such as:


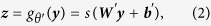


with 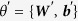 and 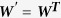 (i.e., tied weights). The hope is that the code ***y*** is a distributed representation that captures the coordinates along the main factors of variation in the data. When training the model, the algorithm searches the parameters that minimize the difference between ***x*** and ***z*** (i.e., the reconstruction error 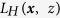).

Autoencoders are often trained to reconstruct the input from a noisy version of the initial data (i.e., denoising) in order to prevent overfitting. This is done by first corrupting the initial input ***x*** to get a partially destroyed version 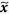 through a stochastic mapping 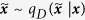. The corrupted input 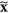 is then mapped, as with the basic autoencoder, to a hidden code 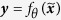 and then to the decoded representation ***z*** (see the Supplementary Appendix A online for a graphical representation). We implemented input corruption using the masking noise algorithm^18^, in which a fraction ***y*** of the elements of ***x*** chosen at random is turned to zero. This can be viewed as simulating the presence of missed components in the EHRs (e.g., medications or diagnoses not recorded in the patient records), thus assuming that the input clinical data is a degraded or “noisy” version of the actual clinical situation. All information about those masked components is then removed from that input pattern, and denoising autoencoders can be seen as trained to fill-in these artificially introduced blanks.

The parameters of the model *θ* and *θ*′ are optimized over the training dataset to minimize the average reconstruction error,


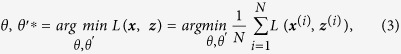


where 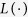 is a loss function and *N* is the number of patients in the training set. We used the reconstruction cross-entropy function as loss function, i.e.,


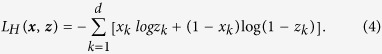


Optimization is carried out by mini-batch stochastic gradient descent, which iterates through small subsets of the training patients and modifies the parameters in the opposite direction of the gradient of the loss function to minimize the reconstruction error. The learned encoding function 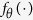 is then applied to the clean input ***x*** and the resulting code ***y*** is the distributed representation (i.e., the input of the following autoencoder in the SDA architecture or the final deep patient representation).

### Evaluation Design

Feature learning algorithms are usually evaluated in supervised applications to take advantage of the available manually annotated labels. Here we used the Mount Sinai data warehouse to learn the deep features and we evaluated them in predicting patient future diseases. The Mount Sinai Health System generates a high volume of structured, semi-structured and unstructured data as part of its healthcare and clinical operations, which include inpatient, outpatient and emergency room visits. Patients in the system can have as long as 12 years of follow up unless they moved or changed insurance. Electronic records were completely implemented by our health system starting in 2003. The data related to patients who visited the hospital prior to 2003 was migrated to the electronic format as well but we may lack certain details of hospital visits (i.e., some diagnoses or medications may not have been recorded or transferred). The entire EHR dataset contains approximately 4.2 million de-identified patients as of March 2015, and it was made available for use under IRB approval following HIPAA guidelines. We retained all patients with at least one diagnosed disease expressed as numerical ICD-9 between 1980 and 2014, inclusive. This led to a dataset of about 1.2 million patients, with every patient having an average of 88.9 records. Then, we considered all records up to December 31, 2013 (i.e., “split-point”) as training data (i.e., 34 years of training information) and all the diagnoses in 2014 as testing data.

### EHR Processing

For each patient in the dataset, we retained some general demographic details (i.e., age, gender and race), and common clinical descriptors available in a structured format such as diagnoses (ICD-9 codes), medications, procedures, and lab tests, as well as free-text clinical notes recorded before the split-point. All the clinical records were pre-processed using the Open Biomedical Annotator to obtain harmonized codes for procedures and lab tests, normalized medications based on brand name and dosages, and to extract clinical concepts from the free-text notes^19^. In particular, the Open Biomedical Annotator and its RESTful API leverages the National Center for Biomedical Ontology (NCBO) BioPortal^20^, which provides a large set of ontologies, including SNOMED-CT, UMLS and RxNorm, to extract biomedical concepts from text and to provide their normalized and standard versions^21^.

The handling of the normalized records differed by data type. For diagnoses, medications, procedures and lab tests, we simply counted the presence of each normalized code in the patient EHRs, aiming to facilitate the modeling of related clinical events. Free-text clinical notes required more sophisticated processing. We applied the tool described in LePendu *et al*.^22^, which allowed identifying the negated tags and those related to family history. A tag that appeared as negated in the note was considered not relevant and discarded^5^. Negated tags were identified using NegEx, a regular expression algorithm that implements several phrases indicating negation, filters out sentences containing phrases that falsely appear to be negation phrases, and limits the scope of the negation phrases^23^. A tag that was related to family history was just flagged as such and differentiated from the directly patient-related tags. We then analyzed similarities in the representation of temporally consecutive notes to remove duplicated information (e.g., notes recorded twice by mistake)^24^.

The parsed notes were further processed to reduce the sparseness of the representation (about 2 million normalized tags were extracted) and to obtain a semantic abstraction of the embedded clinical information. To this aim we modeled the parsed notes using topic modeling^25^, an unsupervised inference process that captures patterns of word co-occurrences within documents to define topics and represent a document as a multinomial over these topics. Topic modeling has been applied to generalize clinical notes and improve automatic processing of patients data in several studies (e.g., see^5^,^26^,^27^,^28^). We used latent Dirichlet allocation as our implementation of topic modeling^29^ and we estimated the number of topics through perplexity analysis over one million random notes. We found that 300 topics obtained the best mathematical generalization; therefore, each note was eventually summarized as a multinomial of 300 topic probabilities. For each patient, we eventually retained one single topic-based representation averaged over all the notes available before the split-point.

### Dataset

All patients with at least one recorded ICD-9 code were split in three independent datasets for evaluation purposes (i.e., every patient appeared in only one dataset). First, we held back 81,214 patients having at least one new ICD-9 diagnosis assigned in 2014 and at least ten records before that. These patients composed validation (i.e., 5,000 patients) and test (i.e., 76,214 patients) sets for the supervised evaluation (i.e., future disease prediction). In particular, all the diagnoses in 2014 were used to evaluate the predictions computed using the patient data recorded before the split-point (i.e., prediction from the patient clinical status). The requirement of having at least ten records per patient was set to ensure that each test case had some minimum of clinical history that could lead to reasonable predictions. We then randomly sampled a subset of 200,000 different patients with at least five records before the split-point to use as training set for the disease prediction experiment.

We used ICD-9 codes to state the diagnosis of a disease to a patient. However, since different codes can refer to the same disease, we mapped the codes to a disease categorization structure used at Mount Sinai, which groups ICD-9s into a vocabulary of 231 general disease definitions^30^. This list was filtered to retain only diseases that had at least 10 training patients and manually polished by a practicing physician to remove all the diseases that could not be predicted from the considered EHR labels alone because related to social behaviors (e.g., HIV) and external life events (e.g., injuries, poisoning), or that were too general (e.g., “other form of cancers”). The final vocabulary included 78 diseases, which are reported in the Supplementary Appendix B online.

Finally, we created the training set for the feature learning algorithms using the remaining patients having at least five records by December 2013. The choice of having at least five records per patient was done to remove some uninformative cases and to decrease the training set size and, consequently, the time of computation. This lead to a dataset composed of 704,587 patients and 60,238 clinical descriptors. Descriptors appearing in more than 80% of patients or present in fewer than five patients were removed from the dataset to avoid biases and noise in the learning process leading to a final vocabulary of 41,072 descriptors. Overall, the raw patient dataset used for feature learning was composed by 200 million non-zero entries (i.e., about 1% of all the entries in the patient-descriptor matrix).

### Patient Representation Learning

SDAs were applied to the dataset of 704,857 patients to derive the deep patient representation. All the feature values in the dataset were first normalized to lie between zero and one to reduce the variance of the data while preserving zero entries. We used the same parameters in all the autoencoders of the deep architecture (regardless the layer) since this configuration usually leads to similar performances as having different parameters for each layer and is easier to evaluate^18^,^31^. In particular, we found that using 500 hidden units per layer and a noise corruption factor 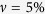 lead to a good generalization error and consistent predictions when tuning the model using the validation data set. We used a deep architecture composed by three layers of autoencoders and sigmoid activation functions (i.e., “DeepPatient”). Preliminary results on disease prediction using a different number of layers are reported in the Supplementary Appendix C online. The deep feature model was then applied to train and test sets for supervised evaluation; hence each patient in these datasets was represented by a dense vector of 500 features.

We compared the deep patient representation with other well-known feature learning algorithms having demonstrated utility in various domains including medicine^12^. All of these algorithms were applied to the scaled dataset as well and performed only one transformation to the original data (i.e., shallow feature learning). In particular, we considered principal component analysis (i.e., “PCA” with 100 principal components), k-means clustering (i.e., “K-Means” with 500 clusters), Gaussian mixture model (i.e., “GMM” with 200 mixtures and full covariance matrix), and independent component analysis (i.e., “ICA” with 100 principal components). In particular, PCA uses an orthogonal transformation to convert a set of observations of possibly correlated variables into a set of linearly uncorrelated variables called principal components, which are less than or equal to the number of original variables. The first principal component accounts for the greatest possible variability in the data, and each succeeding component in turn has the highest variance possible under the constraint that it is orthogonal to the preceding components. K-means groups unlabeled data into *k* clusters, in such a way that each data point belongs to the cluster with the closest mean. In feature learning, the centroids of the cluster are used to produce features, i.e., each feature value is the distance of the data point from each cluster centroid. GMM is a probabilistic model that assumes all the data points are generated from a mixture of a finite number of Gaussian distributions with unknown parameters. ICA represents data using a weighted sum of independent non-Gaussian components, which are learned from the data using signal separation algorithms. As done for DeepPatient, the number of latent variables of each model was identified through preliminary experiments by optimizing learning errors or expectations as well as prediction results obtained in the validation set. We also included in the comparison the patient representation based on the original descriptors after removal of the frequent and rare variables (i.e., “RawFeat” with 41,072 entries).

### Future Disease Prediction

To predict the probability that patients might develop a certain disease given their current clinical status, we implemented random forest classifiers trained over each disease using a dataset of 200,000 patients (*one-vs.-all* learning). We used random forests because they often demonstrate better performances than other standard classifiers, are easy to tune, and are robust to overfitting^32^,^33^. By preliminary experiments on the validation dataset we tuned every disease classifier to have 100 trees. For each patient in the test set (and for all the different representations), we computed the probability to develop every disease in the vocabulary (i.e., each patient was represented by a vector of disease probabilities).

## Results

We evaluated the disease predictions in two applicative clinical tasks: disease classification (i.e., *evaluation by disease*) and patient disease tagging (i.e., *evaluation by patient*). For each patient we considered only the prediction of novel diseases, discarding the re-diagnosis of a disease. If not reported otherwise, all the metrics used in the experiments were upper-bounded by one.

### Evaluation by Disease

To measure how well the deep patient representation performed at predicting whether a patient developed new diseases, we evaluated the ability of the classifier to determine if test patients were likely to be diagnosed with a certain disease within a one-year interval. For each disease, we took the scores obtained by all patients in the test set (i.e., 76,214 patients) and measured the area under the receiver operating characteristic curve (i.e., AUC-ROC), accuracy, and F-score^34^. The ROC curve is a plot of true positive rate versus false positive rate found over the set of predictions. AUC is computed by integrating the ROC curve and it is lower bounded by 0.5. Accuracy is the proportion of true results (both true positives and true negative) among the total number of cases examined. F-score is the harmonic mean of classification precision and recall, where precision is the number of correct positive results divided by the number of all positive results, and recall is the number of correct positive results divided by the number of positive results that should have been returned. Accuracy and F-score require a threshold to discriminate between positive and negative predictions; we set that threshold to 0.6, with this value optimizing the tradeoff between precision and recall for all representations in the validation set by reducing the number of false positive predictions.

The results for all the different data representations are reported in Table 1. The performance metrics of DeepPatient are superior to those obtained by RawFeat (i.e., no feature learning applied to EHR data). In particular, DeepPatient achieved an average AUC-ROC of 0.773, while RawFeat just got 0.659 (i.e., 15% improvement). Accuracy and F-score improved by 15% and 54% respectively, showing that the quality of the positive predictions (i.e., the patients that actually develop that disease) is improved by pre-processing EHRs with a deep architecture. Moreover, DeepPatient consistently and significantly outperforms all other feature learning methods. Table 2 compares the AUC-ROC obtained by RawFeat, PCA and DeepPatient for a subset of 10 diseases (see the Supplementary Appendix D online for the results on the entire vocabulary of diseases). While DeepPatient always outperforms RawFeat, PCA does not lead to any improvement for several diseases (e.g., “Schizophrenia”, “Multiple Myeloma”). Overall, DeepPatient reported the highest AUC-ROC score on every disease but “Cancer of brain and nervous system”, where PCA performed slightly better (AUC-ROC of 0.757 vs. 0.742). Remarkably large improvements in the AUC-ROC score (i.e., more than 60%) were obtained for several diseases, such as “Cancer of testis”, “Attention-deficit and disruptive behavior disorders”, “Sickle cell anemia”, and “Cancer of prostate”. In contrast, some diseases (e.g., “Hypertension”, “Diabetes mellitus without complications”, “Disorders of lipid metabolism”) were difficult to classify and resulted in AUC-ROC scores lower than 0.600 for all representations.

### Evaluation by Patient

**Figure 3:**
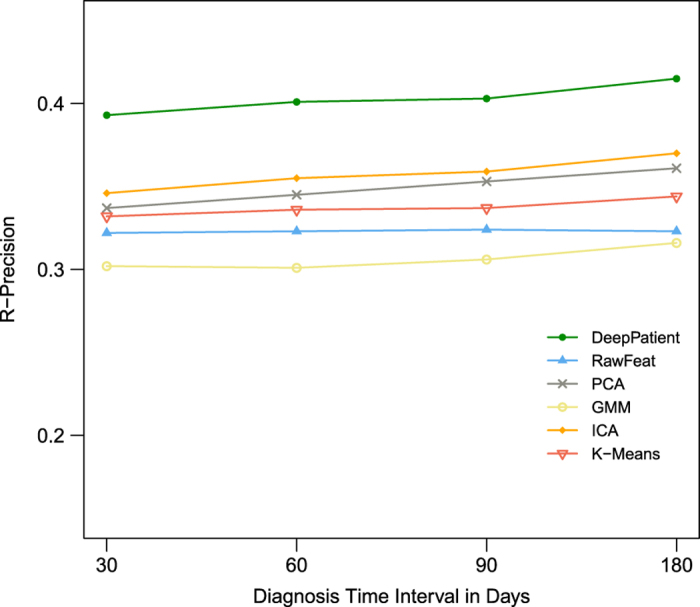
R-precision obtained in the disease tagging experiment by the different patient representations over several prediction time intervals (expressed as number of days). We reports results for patients represented with original descriptors (RawFeat) and pre-processed by principal component analysis (PCA), independent component analysis (ICA), Gaussian mixture model (GMM), k-means clustering (K-Means), and three-layer stacked denoising autoencoders (DeepPatient).

In this experiment we examined how well DeepPatient performed at the patient-specific level. To this aim we retained again only the disease predictions with score greater than 0.6 (i.e., tags) and measured the quality of these annotations over different temporal windows for all the patients having true diagnoses in that period. In particular, we considered diagnoses assigned within 30 (i.e., 16,374 patients), 60 (i.e., 21,924 patients), 90 (i.e., 25,220 patients), and 180 (i.e., 33,607 patients) days. Overall, we found that DeepPatient consistently out-performed other methods across all time intervals examined (Table 3 and Fig. 3).

In particular, we first measured precision-at-*k* (Prec@k, with *k* equal to 1, 3, and 5), which averages the ratio of correct diseases assigned to each patients in each time window within the greatest *k* disease scores (Table 3). In each comparison, we included the model of theoretical upper bound (i.e., “UppBnd”), which reports the best results possible (i.e., all the correct diseases are assigned to each patients). As can be seen, DeepPatient obtained about 55% corrected predictions when suggesting three or more diseases per patient, regardless the time interval. Moreover, when we contrasted DeepPatient with the upper bound, we found a 5–15% improvement over every other method across all times. Last, we report R-precision, which is the precision-at-*R* of the assigned diseases, where *R* is the number of patient diagnoses in the ground truth for the considered time interval^34^ (Fig. 3). Also in this case DeepPatient obtained significant improvements ranging from 5% to 12% over the other models (with ICA obtaining the second best results).

## Discussion

We present a novel application of deep learning to derive predictive patient descriptors from EHRs that we call “deep patient”. This method captures hierarchical regularities and dependencies in the data to create a compact, general-purpose set of patient features that can be effectively used in predictive clinical applications. Results obtained on future disease prediction, in fact, were consistently better than those obtained by other feature learning models as well as than just using the raw EHR data (i.e., the common approach when applying machine learning to EHRs). This shows that pre-processing patient data using a deep sequence of non-linear transformations helps the machine to better understand the information embedded in the EHRs and to effectively make inference out of it. This opens new possibilities for clinical predictive modeling because pre-processing EHR data with deep learning can help improving also ad-hoc frameworks previously proposed in literature towards more effective predictions. In addition, the deep patient leads to more compact and lower dimensional representations than the original EHRs, allowing clinical analytics engines to scale better with the continuous growth of hospital data warehouses.

### Context and Significance

Deep learning was recently applied to medicine and genomics to reconstruct brain circuits^35^ and to predict the activity of potential drug molecules^36^, the effects of mutations in non-coding DNA on gene expressions^37^,^38^, and the sequence specificities of DNA and RNA-binding proteins^39^. To the best of our knowledge, deep feature learning is not yet applied to derive a general-purpose representation of patients from aggregated EHR data. Deep belief networks were recently applied to a small clinical dataset of Chinese patients to recommend acupuncture treatments, with features that were supervised optimized for the specific task^40^. Differently, we applied deep learning to derive patient representations from a large-scale dataset that are not optimized for any specific task and can fit different clinical applications.

We used stacked denoising autoencoders (SDAs) to process EHR data and learn the deep patient representation. SDAs are sequences of three-layer neural networks with a central layer to reconstruct high-dimensional input vectors^12^,^17^,^18^,^41^. To the best of our knowledge, SDAs have never been applied in the clinical domain. A two-layer stacked autoencoder without the denoising component was applied to EHRs to model longitudinal sequences of serum uric acid measurements in order to suggest multiple population subtypes and to distinguish the uric-acid signatures of gout vs. acute leukemia despite not being optimized for the task^42^. Here we apply SDAs and feature learning to derive a general representation of the patients, without focusing on a particular clinical descriptor or domain.

The deep patient representation was evaluated by predicting patient’s future diseases—modeling a practical task in clinical decision making. Previous studies investigated disease prediction in several specific domains, including cardiovascular disease^43^, heart failure^9^, bone diseases^44^, chronic kidney disease^45^, as well as for diagnosis code assignment^46^,^47^. However, the previous efforts generally develop approaches that are highly tuned to a specific disease or phenotype. In contrast, we focused the evaluation of our method on different diseases to show that the deep patient framework learns descriptors that are not domain specific.

### Potential Applications

The deep patient representation improved predictions for different categories of diseases. This demonstrates that the learned features describe patients in a way that is general and effective to be processed by automated methods in different domains. We believe that a deep patient representation inferred from EHRs could benefit other tasks as well, such as personalized prescriptions, treatment recommendations, and clinical trial recruitment. In contrast to representations that are supervised optimized for a specific task^48^, a completely unsupervised vector-oriented representation can be applied to other unsupervised tasks as well, such as patient clustering and similarity. This work represents a first step towards the next generation of predictive clinical systems that can (i) scale to include many millions to billions of patient records and (ii) use a single, distributed patient representation to effectively support clinicians in their daily activities—rather than multiple systems working with different patient representations. In this scenario, the deep learning framework would be deployed to the EHR system and models would be constantly updated to follow the changes in the patient population. However, given that the feature learned by neural networks are not easily interpretable, the framework would be paired with a feature selection tools to help the clinicians understanding what drove the different predictions.

Higher-level descriptors derived from a large-scale patient data warehouse can also enhance the sharing of information between hospitals. In fact, deep features can abstract patient data to a higher level that cannot be fully reconstructed, which facilitates the safe exchange of data between institutions to derive additional representations based on different population distributions (provided with the same underlying EHR representation). As an example, a patient having a clinical status not common for the area where he resides could benefit from being represented using features learned from other hospital data warehouses, where his conditions might be more common. In addition, collaboration between hospitals towards a joint feature learning effort would lead to even better deep representations that would likely improve the design and the performances of a large number of healthcare analytics platforms.

The disease prediction application that was evaluated in this study can be used in a number of clinical tasks towards personalized medicine, such as data-driven assessment of individual patient risk. In fact, clinicians could benefit from a healthcare platform that learns optimal care pathways from the historical patient data, which is a natural extension of the deep patient approach. For example, physicians could monitor their patients, check if any disease is likely to occur in the near future given the clinical status, and preempt the trajectory through data driven selection of interventions. Similarly, the platform could automatically detect patients of the hospital with high probability to develop certain diseases and alert the appropriate care providers.

### Limitations and Future Works

We note some limitations of the current study that highlight opportunities for future method enhancement. As already mentioned, some diseases did not show high predictive power. This was partially related to the fact that we only included the frequency of a laboratory test and we relied on test co-occurrences to determine patient patterns, but we did not considered the test result. Yet, lab test results are not easy to process at this large scale, since they can be available as text flags, values with different unit of measure, ranges, and so on. However, we found that some of the diseases with low performance metrics (e.g., “Diabetes mellitus without complications”, “Hypertension”) are usually screened by laboratory tests collected during routine checkups, making the frequency of those tests not valid discriminant factors. Future work will explore how to include the lab test values to improve the performance of the deep patient representation (i.e., better raw representations are likely to lead to better deep models). Similarly, describing a patient with a temporal sequence of vectors covering predefined consecutive time intervals instead of summarizing all data in one vector is expected to improve the final result as well. The addition of other categories of EHR data, such as insurance details, family history and social behaviors, might also lead to better representations that should obtain reliable prediction models in a larger number of clinical domains. Moreover, the SDA model is likely to take benefit of additional data pre-processing. A common extension is to pre-process the data using PCA to remove irrelevant factors before deep modeling^31^. This approach improved both accuracy and efficiency with other media and should benefit the clinical domain as well.

In future work we plan to investigate the application of the deep representations to other clinical tasks involving automatic prediction, such as personalized prescriptions, therapy recommendation, and clinical trial recruitment. We also plan to investigate thoroughly the application of the deep patient to a specific clinical domain and task to qualitatively evaluate its outcomes (e.g., what are the rules the algorithm discovers and that improve the predictions, how they can be visualized, if they are novel). Further, we aim to evaluate the methodology on the EHR data warehouse of other institutions to consolidate the results as well as to improve the learned features that will benefit from being estimated over a larger number of patients.

## Additional Information

**How to cite this article**: Miotto, R. *et al*. Deep Patient: An Unsupervised Representation to Predict the Future of Patients from the Electronic Health Records. *Sci. Rep.* **6**, 26094; doi: 10.1038/srep26094 (2016).

## Supplementary Material

Supplementary Information

## Figures and Tables

**Table 1:** Disease classification results in terms of area under the ROC curve (AUC-ROC), accuracy and F-score.

Time Interval = 1 year (76,214 patients)			
Patient Representation	AUC-ROC	Classification Threshold = 0.6	
		Accuracy	F-Score
RawFeat	0.659	0.805	0.084
PCA	0.696	0.879	0.104
GMM	0.632	0.891	0.072
K-Means	0.672	0.887	0.093
ICA	0.695	0.882	0.101
DeepPatient	0.773^*^	0.929^*^	0.181^*^

(*) The difference with the corresponding second best measurement is statistically significant (p < 0.05, t-test).

**Table 2:** Area under the ROC curve obtained in the disease classification experiment using patient data represented with original descriptors (“RawFeat”) and pre-processed by principal component analysis (“PCA”) and three-layer stacked denoising autoencoders (“DeepPatient”).

Time Interval = 1 year (76,214 patients)			
Disease	Area under the ROC curve		
	RawFeat	PCA	DeepPatient
Diabetes mellitus with complications	0.794	0.861	**0.907**
Cancer of rectum and anus	0.863	0.821	**0.887**
Cancer of liver and intrahepatic bile duct	0.830	0.867	**0.886**
Regional enteritis and ulcerative colitis	0.814	0.843	**0.870**
Congestive heart failure (non-hypertensive)	0.808	0.808	**0.865**
Attention-deficit and disruptive behavior disorders	0.730	0.797	**0.863**
Cancer of prostate	0.692	0.820	**0.859**
Schizophrenia	0.791	0.788	**0.853**
Multiple myeloma	0.783	0.739	**0.849**
Acute myocardial infarction	0.771	0.775	**0.847**

**Table 3:** Patient disease tagging results for diagnoses assigned during different time intervals in terms of precision-at-*k*, with *k* **= **1, 3, 5; UppBnd shows the best results achievable (i.e., all the correct diagnoses assigned to all the patients).

Time Interval	Metrics	UppBnd	Patient Representation			
			RawFeat	PCA	ICA	DeepPatient
**30 days** *(16,374 patients)*	Prec@1	1.000	0.319	0.343	0.345	0.392^*^
	Prec@3	0.492	0.217	0.251	0.255	0.277^*^
	Prec@5	0.319	0.191	0.214	0.215	0.226^*^
**60 days** *(21,924 patients)*	Prec@1	1.000	0.329	0.349	0.353	0.402^*^
	Prec@3	0.511	0.221	0.254	0.259	0.282^*^
	Prec@5	0.335	0.199	0.216	0.219	0.230^*^
**90 days** *(25,220 patients)*	Prec@1	1.000	0.332	0.353	0.360	0.404^*^
	Prec@3	0.521	0.243	0.257	0.262	0.285^*^
	Prec@5	0.345	0.201	0.219	0.220	0.232^*^
**180 days** *(33,607 patients)*	Prec@1	1.000	0.331	0.361	0.363	0.418^*^
	Prec@3	0.549	0.246	0.261	0.265	0.290^*^
	Prec@5	0.370	0.207	0.221	0.224	0.236^*^

(*) The difference with the corresponding second best measurement is statistically significant (p < 0.05, t-test).

## References

[b1] Hersh W. R. Adding value to the electronic health record through secondary use of data for quality assurance, research, and surveillance. Am. J. Manag. Care 13, 277–278 (2007). 17567224

[b2] Tatonetti N. P., Ye P. P., Daneshjou R. & Altman R. B. Data-driven prediction of drug effects and interactions. Sci. Transl. Med. 4, 125ra131 (2012). 10.1126/scitranslmed.3003377 PMC3382018 22422992

[b3] Li L. . Identification of type 2 diabetes subgroups through topological analysis of patient similarity. Sci. Transl. Med. 7, 311ra174 (2015). 10.1126/scitranslmed.aaa9364 PMC4780757 26511511

[b4] Doshi-Velez F., Ge Y. & Kohane I. Comorbidity clusters in autism spectrum disorders: an electronic health record time-series analysis. Pediatrics 133, e54–63 (2014). 24323995 10.1542/peds.2013-0819 PMC3876178

[b5] Miotto R. & Weng C. Case-based reasoning using electronic health records efficiently identifies eligible patients for clinical trials. J. Am. Med. Inform. Assoc. 22, E141–E150 (2015). 25769682 10.1093/jamia/ocu050 PMC4428438

[b6] Bellazzi R. & Zupan B. Predictive data mining in clinical medicine: current issues and guidelines. Int. J. Med. Inform. 77, 81–97 (2008). 17188928 10.1016/j.ijmedinf.2006.11.006

[b7] Jensen P. B., Jensen L. J. & Brunak S. Mining electronic health records: towards better research applications and clinical care. Nat. Rev. Genet. 13, 395–405 (2012). 22549152 10.1038/nrg3208

[b8] Dahlem D., Maniloff D. & Ratti C. Predictability bounds of electronic health records. Sci. Rep. 5, 11865 (2015). 26148751 10.1038/srep11865 PMC4493571

[b9] Wu J. L., Roy J. & Stewart W. F. Prediction modeling using EHR data: challenges, strategies, and a comparison of machine learning approaches. Med. Care 48, S106–S113 (2010). 20473190 10.1097/MLR.0b013e3181de9e17

[b10] Weiskopf N. G., Hripcsak G., Swaminathan S. & Weng C. Defining and measuring completeness of electronic health records for secondary use. J. Biomed. Inform. 46, 830–836 (2013). 23820016 10.1016/j.jbi.2013.06.010 PMC3810243

[b11] Weiskopf N. G. & Weng C. Methods and dimensions of electronic health record data quality assessment: enabling reuse for clinical research. J. Am. Med. Inform. Assoc. 20, 144–151 (2013). 22733976 10.1136/amiajnl-2011-000681 PMC3555312

[b12] Bengio Y., Courville A. & Vincent P. Representation learning: a review and new perspectives. IEEE T. Pattern Anal. Mach. Intell. 35, 1798–1828 (2013). 10.1109/TPAMI.2013.50 23787338

[b13] Jordan M. I. & Mitchell T. M. Machine learning: trends, perspectives, and prospects. Science 349, 255–260 (2015). 26185243 10.1126/science.aaa8415

[b14] Huang S. H. . Toward personalizing treatment for depression: predicting diagnosis and severity. J. Am. Med. Inform. Assoc. 21, 1069–1075 (2014). 24988898 10.1136/amiajnl-2014-002733 PMC4215055

[b15] Lyalina S. . Identifying phenotypic signatures of neuropsychiatric disorders from electronic medical records. J. Am. Med. Inform. Assoc. 20, e297–305 (2013). 23956017 10.1136/amiajnl-2013-001933 PMC3861917

[b16] Wang X., Sontag D. & Wang F. Unsupervised learning of disease progression models. ACM SIGKDD, 85–94 (2014).

[b17] LeCun Y., Bengio Y. & Hinton G. Deep learning. Nature 521, 436–444 (2015). 26017442 10.1038/nature14539

[b18] Vincent P., Larochelle H., Lajoie I., Bengio Y. & Manzagol P. A. Stacked denoising autoencoders: learning useful representations in a deep network with a local denoising criterion. J. Mach. Learn. Res. 11, 3371–3408 (2010).

[b19] Shah N. H. . Comparison of concept recognizers for building the Open Biomedical Annotator. BMC Bioinformatics 10, S14 (2009). 19761568 10.1186/1471-2105-10-S9-S14 PMC2745685

[b20] Musen M. A. . The National Center for Biomedical Ontology. J. Am. Med. Inform. Assoc. 19, 190–195 (2012). 22081220 10.1136/amiajnl-2011-000523 PMC3277625

[b21] Jonquet C., Shah N. H. & Musen M. A. The Open Biomedical Annotator. Summit on Translat. Bioinforma. 2009, 56–60 (2009). 21347171 PMC3041576

[b22] Lependu P., Iyer S. V., Fairon C. & Shah N. H. Annotation analysis for testing drug safety signals using unstructured clinical notes. J. Biomed. Semantics 3, S5 (2012). 22541596 10.1186/2041-1480-3-S1-S5 PMC3337270

[b23] Chapman W. W., Bridewell W., Hanbury P., Cooper G. F. & Buchanan B. G. A simple algorithm for identifying negated findings and diseases in discharge summaries. J. Biomed. Inform. 34, 301–310 (2001). 12123149 10.1006/jbin.2001.1029

[b24] Cohen R., Elhadad M. & Elhadad N. Redundancy in electronic health record corpora: analysis, impact on text mining performance and mitigation strategies. BMC Bioinformatics 14, 10 (2013). 23323800 10.1186/1471-2105-14-10 PMC3599108

[b25] Blei D. M. Probabilistic topic models. Commun. ACM 55, 77–84 (2012).

[b26] Arnold C. W., El-Saden S. M., Bui A. A. & Taira R. Clinical case-based retrieval using latent topic analysis. AMIA Annu. Symp. Proc., 26–30 (2010). 21346934 PMC3041464

[b27] Perotte A., Bartlett N., Elhadad N. & Wood F. Hierarchically supervised latent dirichlet allocation. NIPS, 2609–2617 (2011).

[b28] Bisgin H., Liu Z., Fang H., Xu X. & Tong W. Mining FDA drug labels using an unsupervised learning technique - topic modeling. BMC Bioinformatics 12, S11 (2011). 22166012 10.1186/1471-2105-12-S10-S11 PMC3236833

[b29] Blei D. M., Ng A. Y. & Jordan M. I. Latent Dirichlet allocation. J. Mach. Learn. Res. 3, 993–1022 (2003).

[b30] Cowen M. E. . Casemix adjustment of managed care claims data using the clinical classification for health policy research method. Med. Care 36, 1108–1113 (1998). 9674627 10.1097/00005650-199807000-00016

[b31] Larochelle H., Bengio Y., Louradour J. & Lamblin P. Exploring strategies for training deep neural networks. J. Mach. Learn. Res. 10, 1–40 (2009).

[b32] Breiman L. Random forests. Mach. Learn. 45, 5–32 (2001).

[b33] Fernandez-Delgado M., Cernadas E., Barro S. & Amorim D. Do we need hundreds of classifiers to solve real world classification problems? J. Mach. Learn. Res. 15, 3133–3181 (2014).

[b34] Manning C. D., Raghavan P. & Schütze H. Introduction to Information Retrieval. (Cambridge University Press, 2008).

[b35] Helmstaedter M. . Connectomic reconstruction of the inner plexiform layer in the mouse retina. Nature 500, 168–174 (2013). 23925239 10.1038/nature12346

[b36] Ma J. S., Sheridan R. P., Liaw A., Dahl G. E. & Svetnik V. Deep neural nets as a method for quantitative structure-activity relationships. J. Chem. Inf. Model 55, 263–274 (2015). 25635324 10.1021/ci500747n

[b37] Leung M. K. K., Xiong H. Y., Lee L. J. & Frey B. J. Deep learning of the tissue-regulated splicing code. Bioinformatics 30, 121–129 (2014). 24931975 10.1093/bioinformatics/btu277 PMC4058935

[b38] Xiong H. Y. . The human splicing code reveals new insights into the genetic determinants of disease. Science 347, 144–151 (2015). 10.1126/science.1254806 PMC4362528 25525159

[b39] Alipanahi B., Delong A., Weirauch M. T. & Frey B. J. Predicting the sequence specificities of DNA- and RNA-binding proteins by deep learning. Nature Biotech. 33, 831–838 (2015). 10.1038/nbt.3300 26213851

[b40] Liang Z., Zhang G., Huang J. X. & Hu Q. V. Deep learning for healthcare decision making with EMRs. IEEE BIBM, 556–559 (2014).

[b41] Hinton G. E. & Salakhutdinov R. R. Reducing the dimensionality of data with neural networks. Science 313, 504–507 (2006). 16873662 10.1126/science.1127647

[b42] Lasko T. A., Denny J. C. & Levy M. A. Computational phenotype discovery using unsupervised feature learning over noisy, sparse, and irregular clinical data. PLoS One 8, e66341 (2013). 23826094 10.1371/journal.pone.0066341 PMC3691199

[b43] Kennedy E. H., Wiitala W. L., Hayward R. A. & Sussman J. B. Improved cardiovascular risk prediction using non-parametric regression and electronic health record data. Med. Care 51, 251–258 (2013). 23269109 10.1097/MLR.0b013e31827da594 PMC4081533

[b44] Hui L., Xiaoyi L., Ramanathan M. & Aidong Z. Prediction and informative risk factor selection of bone diseases. IEEE/ACM T. Comput. Biol. Bioinform. 12, 79–91 (2015). 10.1109/TCBB.2014.2330579 26357080

[b45] Perotte A., Ranganath R., Hirsch J. S., Blei D. & Elhadad N. Risk prediction for chronic kidney disease progression using heterogeneous electronic health record data and time series analysis. J. Am. Med. Inform. Assoc. 22, 872–880 (2015). 25896647 10.1093/jamia/ocv024 PMC4482276

[b46] Perotte A. . Diagnosis code assignment: Models and evaluation metrics. J. Am. Med. Inform. Assoc. 21, 231–237 (2014). 24296907 10.1136/amiajnl-2013-002159 PMC3932472

[b47] Gottlieb A., Stein G. Y., Ruppin E., Altman R. B. & Sharan R. A method for inferring medical diagnoses from patient similarities. BMC Med. 11, 194–203 (2013). 24004670 10.1186/1741-7015-11-194 PMC3844462

[b48] Yao L. X., Zhang Y. Y., Li Y., Sanseau P. & Agarwal P. Electronic health records: Implications for drug discovery. Drug Discov. Today 16, 594–599 (2011). 21624499 10.1016/j.drudis.2011.05.009

