# Supplementary material for: Deep Patient: An Unsupervised Representation to Predict the Future of Patients from the Electronic Health Records
Source: Sci Rep. 2016 May 17;6:26094. doi: 10.1038/srep26094 (PMC4869115; doi:10.1038/srep26094)
Supplement: Supplementary Information [file srep26094-s1.pdf]

SUPPLEMENTARY MATERIAL

Deep Patient: An Unsupervised Representation to Predict the Future of Patients from the Electronic Health Records

Riccardo Miotto[1,2], Li Li[1,2], Brian A. Kidd[1,2], and Joel T. Dudley[1,2]

(1) Department of Genetics and Genomic Sciences;
(2) The Harris Center for Precision Wellness;
Icahn School of Medicine at Mount Sinai
770 Lexington Avenue, 15th Floor, New York, NY 10065, USA

APPENDIX A

Denoising Autoencoders: A Graphical Representation
page 2

APPENDIX B

Patient Representation Evaluation: Vocabulary of Diseases
page 3

APPENDIX C

Deep Patient: Evaluation of the Number of Layers in the Deep Architecture
page 5

APPENDIX D

Deep Patient: Disease Classification Results
page 6

APPENDIX A

Denoising Autoencoders: A Graphical Representation

We report a graphical overview of the denoising autoencoder architecture described in the paper. An example $\boldsymbol{x}$ is stochastically corrupted by q_D (implemented as masking noise corruption) to $\tilde{\boldsymbol{x}}$. The autoencoder then maps $\tilde{\boldsymbol{x}}$ to $\boldsymbol{y}$ using the *encoder* $f_\theta(\cdot)$ and attempts to reconstruct $\boldsymbol{x}$ with the *decoder* $g_{\theta'}(\cdot)$, obtaining $\boldsymbol{z}$. When training the model, the difference between $\boldsymbol{x}$ and $\boldsymbol{z}$, which is minimized using the stochastic gradient descent algorithm, is measured by the loss function $L_H(\boldsymbol{x}, \boldsymbol{z})$. In this study we used the reconstruction cross-entropy as loss function. The learned encoding $f_\theta(\cdot)$ function is then applied to the original input $\boldsymbol{x}$ to obtain the distributed coded representation.

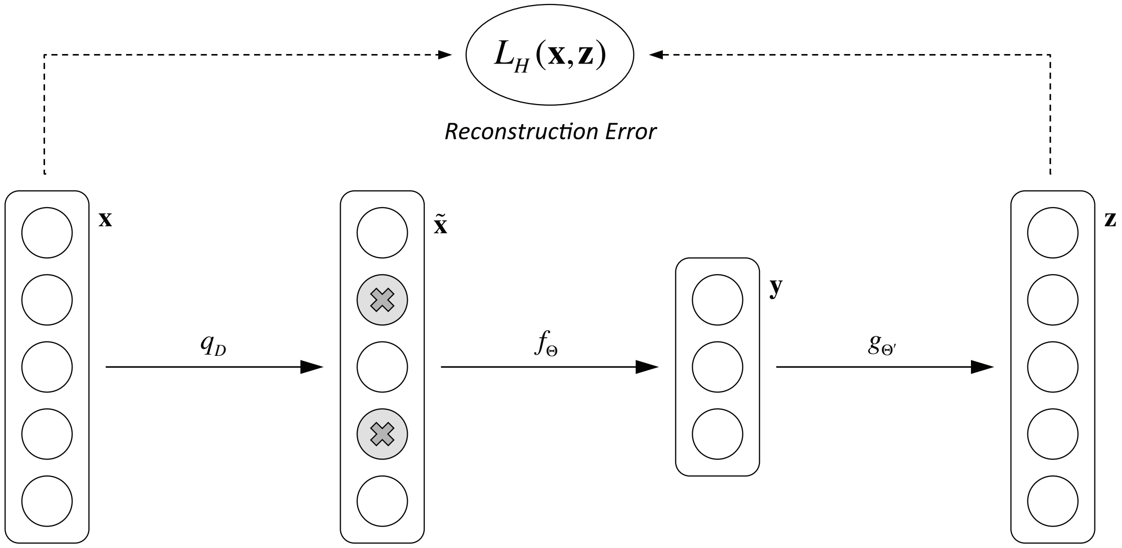

APPENDIX B

Patient Representation Evaluation: Vocabulary of Diseases

List of the 78 diseases, sorted by category, used to evaluate the deep patient representation.

**Category**	**Disease**
Diseases of the blood and blood-forming organs	Coagulation and hemorrhagic disorders<br>Deficiency and other anemia<br>Diseases of white blood cells<br>Sickle cell anemia
Diseases of the circulatory system	Acute cerebrovascular disease<br>Acute myocardial infarction<br>Aortic and peripheral arterial embolism or thrombosis<br>Aortic, peripheral and visceral artery aneurysms<br>Cardiac arrest and ventricular fibrillation<br>Cardiac dysrhythmias<br>Conduction disorders<br>Congestive heart failure (non-hypertensive)<br>Coronary atherosclerosis<br>Heart valve disorders<br>Hypertension<br>Occlusion or stenosis of pre-cerebral arteries<br>Peripheral and visceral atherosclerosis<br>Phlebitis, thrombophlebitis and thromboembolism<br>Pulmonary heart disease
Diseases of the digestive system	Biliary tract disease<br>Diverticulosis and diverticulitis<br>Gastritis and duodenitis<br>Gastroduodenal ulcer (except hemorrhage)<br>Gastrointestinal hemorrhage<br>Intestinal infections<br>Intestinal obstruction without hernia<br>Peritonitis and intestinal abscess<br>Regional enteritis and ulcerative colitis
Diseases of the genitourinary system	Acute and unspecified renal failure<br>Chronic kidney disease<br>Endometriosis<br>Inflammatory conditions of male genital organs<br>Inflammatory diseases of female pelvic organs<br>Menopausal disorders<br>Nephritis, nephrosis and renal sclerosis<br>Prolapse of female genital organs
Diseases of the musculoskeletal system and connective tissue	Osteoarthritis<br>Osteoporosis<br>Spondylosis and intervertebral disc disorders

Diseases of the nervous system and sense organs	Glaucoma<br>Parkinson`s disease<br>Retinal detachments and retinopathy
Diseases of the respiratory system	Chronic obstructive pulmonary disease and bronchiectasis<br>Pleurisy, pneumothorax and pulmonary collapse<br>Respiratory failure, insufficiency and arrest
Endocrine, nutritional and metabolic diseases and immunity disorders	Disorders of lipid metabolism<br>Diabetes mellitus with complications<br>Diabetes mellitus without complications<br>Gout and other crystal arthropathies<br>Immunity disorders<br>Thyroid disorders
Mental Illness	Adjustment disorders<br>Alcohol-related disorders<br>Anxiety disorders<br>Attention-deficit and disruptive behavior disorders<br>Delirium, dementia and amnestic (and other) cognitive disorders<br>Developmental disorders<br>Mood disorders<br>Personality disorders<br>Schizophrenia
Neoplasms	Cancer of bladder<br>Cancer of brain and nervous system<br>Cancer of breast<br>Cancer of bronchus and lung<br>Cancer of cervix<br>Cancer of colon<br>Cancer of kidney and renal pelvis<br>Cancer of liver and intrahepatic bile duct<br>Cancer of ovary<br>Cancer of pancreas<br>Cancer of prostate<br>Cancer of rectum and anus<br>Cancer of testis<br>Cancer of uterus<br>Hodgkin`s disease<br>Leukemias<br>Multiple myeloma<br>Non-Hodgkin`s lymphoma

APPENDIX C

Deep Patient: Evaluation of the Number of Layers in the Deep Architecture

We describe the effects of the number of layers (i.e., denoising autoencoders) used to derive the deep representation on the future disease classification results (one-year time interval). The experiment used the same setting described in the paper. In particular, classification models were trained over 200,000 patients and 78 diseases, while the evaluation included 76,214 different patients. The figure below reports accuracy, area under the ROC curve (i.e., AUC-ROC) and F-score, with classification threshold value for accuracy and F-score set to 0.6. The first measure (i.e., number of layers equal to 0) means that feature learning was not applied and classification was performed on the original patient data (i.e., "RawFeat"). As it can be seen, after using three layers results stabilize for all metrics, without leading to any further improvement. For this reason the experiments reported in the paper only included a three-layer deep architecture, which we referred to as "DeepPatient".

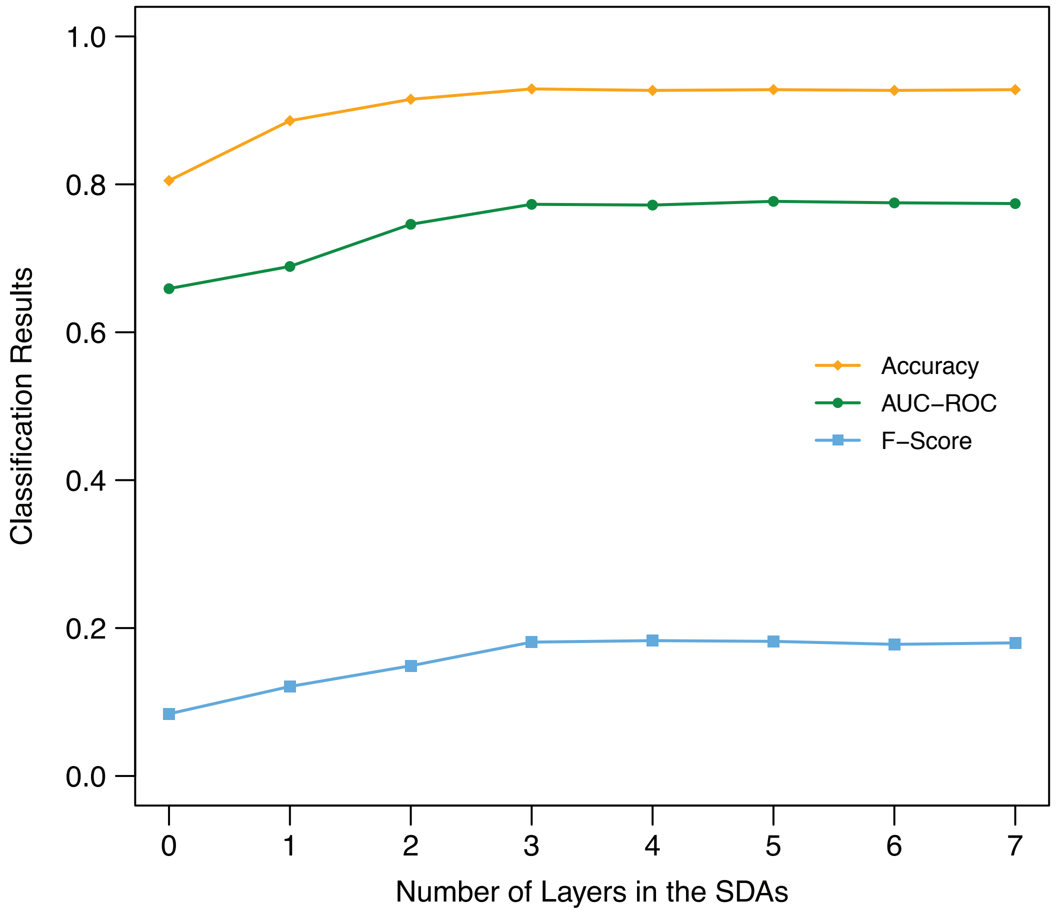

APPENDIX D

Deep Patient: Disease Classification Results

We present the results for all the 78 diseases in the *evaluation by disease* experiment (one-year time interval). In particular we report the area under the ROC curve (i.e., AUC-ROC) obtained using patient data represented with original descriptors ("RawFeat") and pre-processed by principal component analysis ("PCA") and three-layer stacked denoising autoencoders ("DeepPatient"). The experiment used the same setting described in the paper and already reported in **Appendix C** of this supplementary material.

**Time Interval = 1 year (76,214 patients)**			
	**Area under the ROC curve**		
**Disease**	**RawFeat**	**PCA**	**DeepPatient**
Diabetes mellitus with complications	0.794	0.861	**0.907**
Cancer of rectum and anus	0.863	0.821	**0.887**
Cancer of liver and intrahepatic bile duct	0.830	0.867	**0.886**
Regional enteritis and ulcerative colitis	0.814	0.843	**0.870**
Congestive heart failure (non-hypertensive)	0.808	0.808	**0.865**
Attention-deficit and disruptive behavior disorders	0.730	0.797	**0.863**
Cancer of prostate	0.692	0.820	**0.859**
Schizophrenia	0.791	0.788	**0.853**
Multiple myeloma	0.783	0.739	**0.849**
Acute myocardial infarction	0.771	0.775	**0.847**
Personality disorders	0.787	0.788	**0.846**
Inflammatory conditions of male genital organs	0.659	0.825	**0.841**
Endometriosis	0.697	0.765	**0.839**
Inflammatory diseases of female pelvic organs	0.714	0.799	**0.830**
Cancer of ovary	0.646	0.788	**0.824**
Sickle cell anemia	0.567	0.689	**0.822**
Nephritis, nephrosis and renal sclerosis	0.763	0.775	**0.821**
Cancer of bladder	0.711	0.744	**0.818**
Chronic kidney disease	0.764	0.758	**0.814**
Cancer of testis	0.508	0.771	**0.811**
Menopausal disorders	0.681	0.772	**0.808**
Delirium, dementia and amnestic (and other) cognitive disorders	0.728	0.720	**0.803**
Peritonitis and intestinal abscess	0.689	0.747	**0.801**
Cardiac arrest and ventricular fibrillation	0.711	0.747	**0.799**
Developmental disorders	0.705	0.737	**0.798**

Cancer of pancreas	0.697	0.595	**0.795**
Respiratory failure, insufficiency and arrest	0.700	0.718	**0.788**
Peripheral and visceral atherosclerosis	0.724	0.741	**0.786**
Coronary atherosclerosis	0.740	0.751	**0.783**
Immunity disorders	0.711	0.681	**0.780**
Acute and unspecified renal failure	0.703	0.730	**0.778**
Intestinal obstruction without hernia	0.686	0.722	**0.775**
Leukemias	0.738	0.708	**0.774**
Cancer of uterus	0.618	0.707	**0.771**
Non-Hodgkin`s lymphoma	0.708	0.676	**0.771**
Cancer of bronchus and lung	0.688	0.702	**0.770**
Cancer of colon	0.696	0.664	**0.767**
Conduction disorders	0.697	0.722	**0.765**
Pulmonary heart disease	0.679	0.710	**0.764**
Aortic, peripheral and visceral artery aneurysms	0.685	0.699	**0.763**
Cancer of breast	0.651	0.697	**0.762**
Prolapse of female genital organs	0.664	0.700	**0.761**
Adjustment disorders	0.693	0.697	**0.757**
Parkinson`s disease	0.665	0.672	**0.754**
Cancer of kidney and renal pelvis	0.679	0.701	**0.753**
Occlusion or stenosis of pre-cerebral arteries	0.664	0.689	**0.752**
Aortic and peripheral arterial embolism or thrombosis	0.652	0.662	**0.752**
Phlebitis, thrombophlebitis and thromboembolism	0.672	0.683	**0.747**
Cancer of brain and nervous system	0.731	**0.757**	0.742
Gout and other crystal arthropathies	0.660	0.681	**0.738**
Acute cerebrovascular disease	0.670	0.681	**0.738**
Retinal detachments and retinopathy	0.680	0.672	**0.737**
Hodgkin`s disease	0.639	0.644	**0.731**
Pleurisy, pneumothorax and pulmonary collapse	0.663	0.677	**0.727**
Osteoarthritis	0.654	0.659	**0.723**
Glaucoma	0.689	0.632	**0.707**
Intestinal infection	0.632	0.608	**0.692**
Mood disorders	0.645	0.650	**0.691**
Coagulation and hemorrhagic disorders	0.641	0.635	**0.688**
Chronic obstructive pulmonary disease and bronchiectasis	0.616	0.612	**0.688**
Biliary tract disease	0.628	0.620	**0.676**
Cancer of cervix	0.586	0.631	**0.675**
Gastroduodenal ulcer (except hemorrhage)	0.612	0.633	**0.674**

Alcohol-related disorders	0.610	0.627	**0.670**
Diseases of white blood cells	0.620	0.635	**0.666**
Gastritis and duodenitis	0.619	0.611	**0.663**
Heart valve disorders	0.596	0.619	**0.660**
Spondylosis and intervertebral disc disorders	0.608	0.602	**0.651**
Diverticulosis and diverticulitis	0.569	0.599	**0.644**
Gastrointestinal hemorrhage	0.608	0.608	**0.640**
Thyroid disorders	0.601	0.613	**0.634**
Osteoporosis	0.541	0.600	**0.626**
Cardiac dysrhythmias	0.565	0.587	**0.609**
Anxiety disorders	0.572	0.564	**0.605**
Deficiency and other anemia	0.567	0.576	**0.603**
Diabetes mellitus without complications	0.564	0.552	**0.586**
Hypertension	0.536	0.528	**0.574**
Disorders of lipid metabolism	0.549	0.527	**0.561**
